# Which Comes First in Sports Vision Training: The Software or the Hardware Update? Utility of Electrophysiological Measures in Monitoring Specialized Visual Training in Youth Athletes

**DOI:** 10.3389/fnhum.2021.732303

**Published:** 2021-10-06

**Authors:** Dmitri Poltavski, David Biberdorf, Carolina Praus Poltavski

**Affiliations:** ^1^Department of Psychology, University of North Dakota, Grand Forks, ND, United States; ^2^Valley Vision Clinic, Grand Forks, ND, United States

**Keywords:** sports vision training, visual software, visual hardware, ice hockey, VEP, EEG, STMLI, Senaptec Sensory Station

## Abstract

In the present study we combined popular methods of sports vision training (SVT) with traditional oculomotor protocols of Optometric Vision Therapy (OVT) and electrophysiological indexes of EEG and VEP activity to monitor training progress and changes in performance of youth ice hockey players without the history of concussion. We hypothesized that administration of OVT protocols before SVT training may result in larger performance improvements compared to the reverse order due to the initial strengthening of visual hardware capable of handling greater demands during training of visuomotor integration and information processing skills (visual software). In a cross-over design 53 youth ice hockey players (ages 13–18) were randomly assigned to one of the two training groups. Group one (hardware-software group) completed 5 weeks of oculomotor training first followed by 5 weeks of software training. For group 2 (software-hardware) the order of procedures were reversed. After 10 weeks of training both groups significantly improved their performance on all but one measure of the Nike/Senaptec Sensory station measures. Additionally, the software-hardware training order resulted in significantly lower frontal theta-to-gamma amplitude ratios on the Nike/Senaptec test of Near-Far Quickness as well as in faster P100 latencies. Both training orders also resulted in significant decreases in post-treatment P100 amplitude to transient VEP stimuli as well as decreased theta-gamma ratios for perception span, Go/No-Go and Hand Reaction time. The observed changes in the electrophysiological indexes in the present study are thought to reflect greater efficiency in visual information processing and cognitive resource allocation following 10 weeks of visual training. There is also some evidence of the greater effectiveness of the software-hardware training order possibly due to the improved preparedness of the oculomotor system in the youth athletes for administration of targeted protocols of the Optometric Vision Therapy.

Which comes first in Sports Vision Training: the software or the hardware update? Utility of electroencephalographic measures in monitoring specialized visual training in youth athletes.

## Introduction

### Literature Review

Over the past several decades, there have been numerous studies involving training or enhancement of normal vision function with the almost unanimous conclusion that most visual functions can be improved by specific laboratory-based vision training paradigms (i.e., repeated practice of some highly specific task; Ciuffreda and Wang, 2004). In their meta-analysis Ciuffreda and Wang (2004) delineated 5 major visual categories thought to be important for sport-specific performance which included static and dynamic visual acuity, depth perception, tracking of moving objects (version and vergence), visuo-motor integration (i.e., eye-hand coordination) and visual information processing (selective attention, anticipation, visual imagery and decision making). The researchers further suggested that training of higher information processing skills (i.e., as prediction/anticipation, recall, cognitive strategy and decision making) may be of central importance to enhanced athletic performance.

Indeed, the decision-making ability seems to be more consistently associated with expert athletic performance compared to novices. In their meta-analysis of 42 studies Mann et al. (2007) concluded that experts were more accurate in their decision making relative to their lesser skilled counterparts and anticipated their opponents' intentions significantly quicker than less skilled participants suggesting that the use of advanced perceptual cues facilitates sport performance by means of aiding in the anticipation of opponent's actions and decreasing overall response time. In another meta-analysis of 20 studies Voss et al. (2010) also found a small-to-moderate effect size for the difference between experts vs. non-experts in multiple sports on basic cognitive measures of visual attention and processing speed.

In addition, modulation of attention is presumably important for the majority of competitive sports (Di Russo et al., 2003), as most sports are not exclusively played at a distance but involve rapid target shifts between far, intermediate, and near distances requiring rapid accommodative-vergence responses (Erickson et al., 2011). Ciuffreda and Wang (2004) went further to suggest that visual attentional training (e.g., dynamically shifting or weighting one's visual attentional focus from one region of the visual field to another) should be incorporated into any sports vision-training paradigm irrespective of a given sport.

As a result, state-of-the-art sports vision programs now employ a variety of digital training programs targeting perceptual-cognitive processes (see Appelbaum and Erickson, 2018 for a review). The most comprehensive of these tools include integrated visual assessment and training systems such as the Nike SPARQ Sensory Training Station and its successor the Senaptec Sensory Station. While these systems test a broad range of basic visual and information processing skills [i.e., static visual acuity, dynamic visual acuity, contrast sensitivity, distance stereopsis, accommodative-vergence facility, central eye-hand reaction and response speeds, peripheral eye-hand proaction, span of perception and stimulus discrimination and multiple object tracking (Senaptec)], their training modules place primary emphasis on visuomotor integration and information processing skills.

In one of our previous studies we used the Nike SST to evaluate visual function of 42 male and female Division I collegiate hockey players. We found that the athletes' scores on such measures as decision-making (Go/No-Go), dynamic depth perception, perception span and reaction time were able to predict 69% of variance in goals scored by these players in the subsequent 2 hockey seasons (Poltavski and Biberdorf, 2015). These findings thus confirmed the importance of focusing sport vision training on visuo-motor and information processing skills. Recently, in a placebo-controlled study Liu et al. (2020) also showed that on average 8.5 h of Dynamic Vision Training (DVT) in 24 Division I collegiate baseball players resulted in a significant sports-related skills transfer in launch angle and hit distance compared to the placebo group. The DVT utilized Senaptic Stroboscopic eyewear for sports-specific drills as well as Senaptec training modules of dynamic vision and oculomotor and anticipatory timing training.

Electrophysiological (e.g., EEG, VEP) measures may prove particularly useful in assessing effects of sports vision training on visual processing. For example, Zwierko et al. (2014) previously reported a significant reduction in the N75 and P100 VEP component latencies in female volleyball players following 2 years of athletic training. Similar changes were not observed in an age-matched control group. The researchers concluded that systematic physical training that required quick stimulus discrimination and selective visual attention improved the speed of early visual processing. At least two groups also showed utility of measuring occipital alpha power in predicting performance in expert rifle shooters (Liu et al., 2018) and elite soccer goaltenders (Jeunet et al., 2020).

It may also be important to evaluate the utility of electrophysiological measures for monitoring visual training progress in the context of training all aspects of the visual system: both visual software and visual hardware. In Abernethy (1986) proposed that the visual system works separately to gather information and then to process information. The suggestion was that the 'hardware' system can be seen as the mechanical and optometric properties of a person's visual system and that the 'software' system can be seen as the analysis, selection, coding and general handling of the visual information during training and competition. Abernethy (1987) further elaborated that there are six optometric skills that make up the hardware system: *static and dynamic visual acuity, depth perception, accommodation, fusion (convergence), color vision, and contrast sensitivity*. Ferreira (2002) listed just seven optometric skills that make up the software system; *eye-hand coordination, eye-body co-ordination, visual adjustability, visual concentration, central-peripheral awareness, visual reaction time, and visualization*. While this hardware/software dichotomy is somewhat arbitrary, generally the hardware factors do tend to relate more to the reception and sensation of visual information whereas software plays a more dominant role in the subsequent perception (Abernethy, 1987).

Research in the perceptual learning literature demonstrates that 'hardware' skills can indeed be improved. Such improvements have been shown in contrast sensitivity (Sowden et al., 2002), depth perception (Sowden et al., 1996), and visual detection (Schoups et al., 1995). When specialized training programs are intended to enhance basic visual perceptual processes (e.g., static and dynamic visual acuity, vergence eye-movements, combined saccadic/accommodative tracking, visual reaction time, peripheral awareness, and visual search) in athletes, some authors also found improvements in sports-related performance of elite shooters (Quevedo et al., 1999), basketball players (Kofsky and Starfield, 1989) and varsity soccer players (McLeod, 1991).

At the same time positive benefits of such training are expected only under the assumption of intact visual “hardware.” In many athletes, however, visual hardware (such as accommodation and vergence) may be compromised due to a previous history of concussion (Thiagarajan et al., 2011; Poltavski and Biberdorf, 2014), which may potentially compromise sports-vision training. Further deficits are also observed in visual signal processing. In one of our previous studies using Visual Evoked Potentials (Poltavski et al., 2017) we were able to show that among patients with Convergence Insufficiency those with a history of concussion were on average about 16 ms slower in processing vertical sinusoidal stimuli (10% contrast, 4 Hz reversal frequency) along the magnocellular pathway (P100 latency) than those without the history of concussion. The P100 amplitude in the group with a history of concussion was also significantly smaller than in the group without any previous history of mTBI. No such differences between the groups were observed for parvocellular stimuli (checkboard patterns of 85% contrast and 2 Hz reversal frequency). These results suggested magnocellular deficits individuals with a history of mTBI independent of their oculomotor deficits.

Similarly, our group also recently reported EEG-based differences related to the “visual software” in athletes with a history of concussion on tasks of visuo-motor control of the Nike Sensory Station (Poltavski et al., 2019). We used a novel measure that we termed a “Short-term Memory Load Index” (STMLI) to express these EEG differences in performance. The index was derived by taking a ratio of relative Theta_3−7*Hz*_ PSD to relative Gamma_30−40*Hz*_ PSD at electrode location Fz according to the International 10–20 system. This index was based on the findings of Kamiński et al. (2011) who reported that the theta/gamma cycle length ratio obtained from electrode Fz (frontal midline) significantly predicted performance on the digit span task, with greater ratios corresponding to better scores. Our amplitude-based theta-to-gamma ratio at Fz thus proved to be a sensitive index of efficiency of visual processing in athletes with a history of concussion on tasks of visuo-motor control of the Nike Sensory Station (Poltavski et al., 2019).

### Present Study

In the present study we attempted to combine popular methods of sports vision training with traditional oculomotor (hardware) protocols of Optometric Vision Therapy. We hypothesized that our electrophysiological indexes of EEG and VEP activity previously used in research with athletes with a history of concussion (Poltavski et al., 2017, 2019) will also be useful to monitor training progress and changes in performance of youth ice hockey players without the history of concussion. We further hypothesized that administration of OVT protocols before software training may result in larger performance improvements compared to the reverse order due to the initial strengthening of the visual hardware capable of handling greater demands during training of visuomotor integration and information processing skills (visual software). The results of the study were thus aimed at developing more effective sports vision training protocols and were hypothesized to help researchers to better understand neural mechanisms of visual training.

## Materials and Methods

### Participants

Eighty-one youth participants (70 males and 11 females, ages 13–18) were recruited from local and public school extra-curricular athletic programs in ice hockey via their respective activities directors and athletic program directors. The study protocols were approved by the Institutional Review Board (IRB) of the University of North Dakota. The participation was voluntary, and the participants had the right to withdraw any time from the study.

Eighteen participants reported having had at least one lifetime concussion (range 1–4, mean 1.83). Eight of these youth athletes reported having had their most recent concussion in the past year, while the remaining 10 indicated having had a concussion over a year before their participation in the study. None of the participants with a history of concussion reported any lasting symptoms and all of them resumed their regular athletic and academic activities following their most recent concussion. Training results of these participants were excluded from the analyses as our previous research as well as study findings of others showed that athletes with a history of concussion may differ from age, gender and sport- matched controls on some of the measures of the Nike Sensory Station (Mihalik and Wasserman, 2017; Poltavski et al., 2019), EEG (Poltavski et al., 2019) and VEP measures (Poltavski et al., 2017). Other exclusionary criteria included previously diagnosed learning disorder(s) such as Attention-Deficit Hyperactivity Disorder and/or dyslexia as well as a number of optometric conditions including strabismus, uncorrected astigmatism and anisometropia, oculomotor deficits such as convergence and accommodative insufficiency as well as significant ocular pathology (excluding color deficiencies). Out of the remaining 63 participants without the history of concussion, 53 completed all 10 weeks of visual training and had all 3 assessments. This was the final sample used in all of the statistical analyses. Participant age range was again between 13 and 18 with a mean age of 13.87 (SD = 1.29) and only 4 participants older than 15. There were 7 girls and 46 boys in the final sample, all ice hockey players from Peewee and Bantam age divisions. There were 11 players with center positions, 6 left wing, 4 right wing, 14 defensemen, and 12 goalies. Six participants did not report their positions.

Furthermore, the training phase of the study lasted for serveral years betweeen 2015 and 2017. Some of the athletes who enrolled in the study in 2015 were tested and trained on the Nike SPARQ Sensory Station, which went offline on October 1st, 2015 and was succeded by the Senaptec Sensory station. The Senaptec Sensory Station was then used for the remainder of the study for both testing and training. Out of the 53 participants included into the analyses 24 completed all their testing on the Nike SPARQ Sensory System while 29 athletes completed all assessments on the Senaptec Sensory station.

### Instruments

#### Assessment Instruments

##### Nike Sensory Performance System and Senaptec Sensory Station

A detailed description of the Nike SPARQ Sensory Performance System (Nike SST) is provided in our earlier paper (Poltavski and Biberdorf, 2015). The Nike SST is a computer-based vision assessment station that evaluates athletes on 9 sport-relevant visual and sensory performance skills. It consists of a single computer controlling two high-resolution liquid crystal display monitors (both 0.2 mm dot pitch): one 22-inch diagonal display and one 42-inch diagonal touch-sensitive display. Custom software controls the displays, input acquisition, and test procedures based on subject responses. Five of the tests are performed 16 feet (4.9 m) from the 22-inch display screen. The subject uses a handheld Apple iPod touch (Apple Corporation, Cuptertino, California), which is connected via wireless input to the computer so that it could interact with the station's screen monitor. These tests include Visual Clarity, Contrast Sensitivity, Depth Perception (Stereopsis) at Far, Near-Far Quickness and Target Capture (dynamic visual acuity). The other 4 tests are performed with the subject positioned within arm's length of the 42-inch touch sensitive screen mounted with the center of the screen at about eye-level. These tests include Perception Span, Eye-Hand Coordination (Peripheral Eye-hand response), Go/No Go and Hand Reaction Time (central eye-hand reaction and response time). Reliability and validity information of the Nike SST output parameters can be found in Erickson et al. (2011). The Nike SST also includes 4 training modules to improve decision making (Go/ No Go), split attention, depth perception and eye-hand coordination.

The Senaptec Sensory Station succeeded the Nike SST in the end of 2015 and besides testing the above 9 visual skills also added Multiple Object Tracking. The Senaptec Sensory Station has also significantly expanded the number of available training modules (15 total). In addition to the original 4 featured in the Nike SST, the Senaptec system also trains dynamic vision, sensory memory, response inhibition, spatial memory, spatial sequence, multiple object tracking, near far shirt, visual search, tempo, shape cancellation and visual motor integration.

##### EEG

In the present study we utilized the B-Alert X10 device (ABM, Carlsbad, California) for wireless EEG recording along 9 channels that collect continuous electroencephalographic signals from electrode locations in the frontal (Fz, F3, and F4), central (Cz, C3, and C4), and parietal-occipital (POz, P3, and P4) areas. These electrode locations are predetermined by distances between sensors on a 9-sensor strip. The size of the strip is selected based on the distance between the subject's nasion and inion. As per manufacturer's recommendation, the medium strip was used for subjects with nasion-inion distances >34.5 cm and the small strip was applied when the distance was between 32.0 and 34.5 cm. Using these guidelines, sensor locations correspond to the International 10–20 system of scalp electrode placement. Disposable self-adhesive sponge electrodes filled with 0.4–0.6 cc of Synapse® conductive electrode cream were attached to the sensor strip before the headset's placement on the participant's scalp. Two mastoid leads with disposable electrodes served as reference and were placed over the participant's mastoid bones on each side of the head.

The B-Alert X-10 has a 256 Hz sampling rate and sends radio signals using a 2.4 to 2.48 GHz radio transmitter to the B-Alert X-10 Live acquisition software that can be run from any PC using a USB receiver. Prior to signal transmission the unit also performs analog-to-digital conversion, encoding, and formatting.

Data were sampled and processed at 256 Hz using a band pass filter from 0.5 to 65 Hz (3 dB attenuation). Notch filters at 50, 60, 100, and 120 Hz were used to specifically target the removal of environmental noise (i.e., electrical interference) not fully attenuated by the band pass filter. The EEG signal was referenced to the linked mastoid-placed electrodes using the average voltage between the reference electrodes. The acquisition software used ABM algorithms to detect and remove artifacts (spikes, excursions, amplifier saturations, electromyography, and eye blinks) prior to power spectral density (PSD) computations. PSD was automatically computed by the B-Alert Live software using a 50% overlapping window across three, 1-s data overlays (256 decontaminated data points each) and applying the Fast Fourier Transformation with Kaiser windowing for data smoothing. If more than 128 zero values were inserted for an overlay, the overlay was excluded from the epoch average; if 2 of the 3 overlays were rejected, the epoch was classified “invalid” excluded from analysis.

This resulted in PSD values for each 1-s epoch for 1 Hz frequency bins ranging from 1 to 40 Hz. PSD values were then log10 transformed by the software to achieve a Gaussian distribution. Relative PSDs were computed automatically by taking the PSD value in the 1 Hz frequency of interest and dividing it by the sum of the PSD values from 1 to 40 Hz. To obtain bandwidth frequencies, the relative 1 Hz PSD bins were averaged across the frequency ranges as follows: 3–7 Hz (theta), 8–12 Hz (alpha), 13–19 Hz (low beta), 20–29 Hz (high beta), and 30–40 Hz (gamma).

A short-term memory load index (STMLI) was then calculated for each participant and each Nike SST task by dividing relative PSD for theta at Fz by the corresponding relative PSD for gamma, resulting in STMLI ratios for each 1-s epoch (see Formula 1).


$$\begin{array}{l} STMLI\;=\frac{rPSD\theta_{Fz}}{rPSD\gamma_{Fz}} \end{array}$$

*Formula 1. Calculation of the Short-term Memory Load Index*

For each Nike SPARQ test, 1-s epochs of STMLI ratios were aggregated using 5% trimmed means for each participant (i.e., mean STMLI ratios were generated for each SPARQ test for each participant). Five percent trimmed means were used to eliminate 1-s epoch with extreme values.

##### VEP Apparatus and Stimuli

Transient VEPs (tVEP) were obtained using a Diopsys NOVA System (Diopsys, Inc., Pine Brook, New Jersey, USA). The stimuli were presented on an Acer V173 43.18 cm LCD monitor (33.7 cm × 27 cm) with a refresh rate of 75 Hz. The stimuli included 2 checkerboard and 2 vertical sine wave gratings.

The checkerboard stimuli were of two sizes: 8 × 8 with 3.38 cm (2-degree check size) and 16 × 16 with 1.69 cm checks (1 degree check size). Both checkerboard patterns had a Michelson contrast of 85%, mean luminance of 102.22 cd/m^2^ and were reversed 2 times per second (temporal frequency 1 Hz).

**Figure 1:**
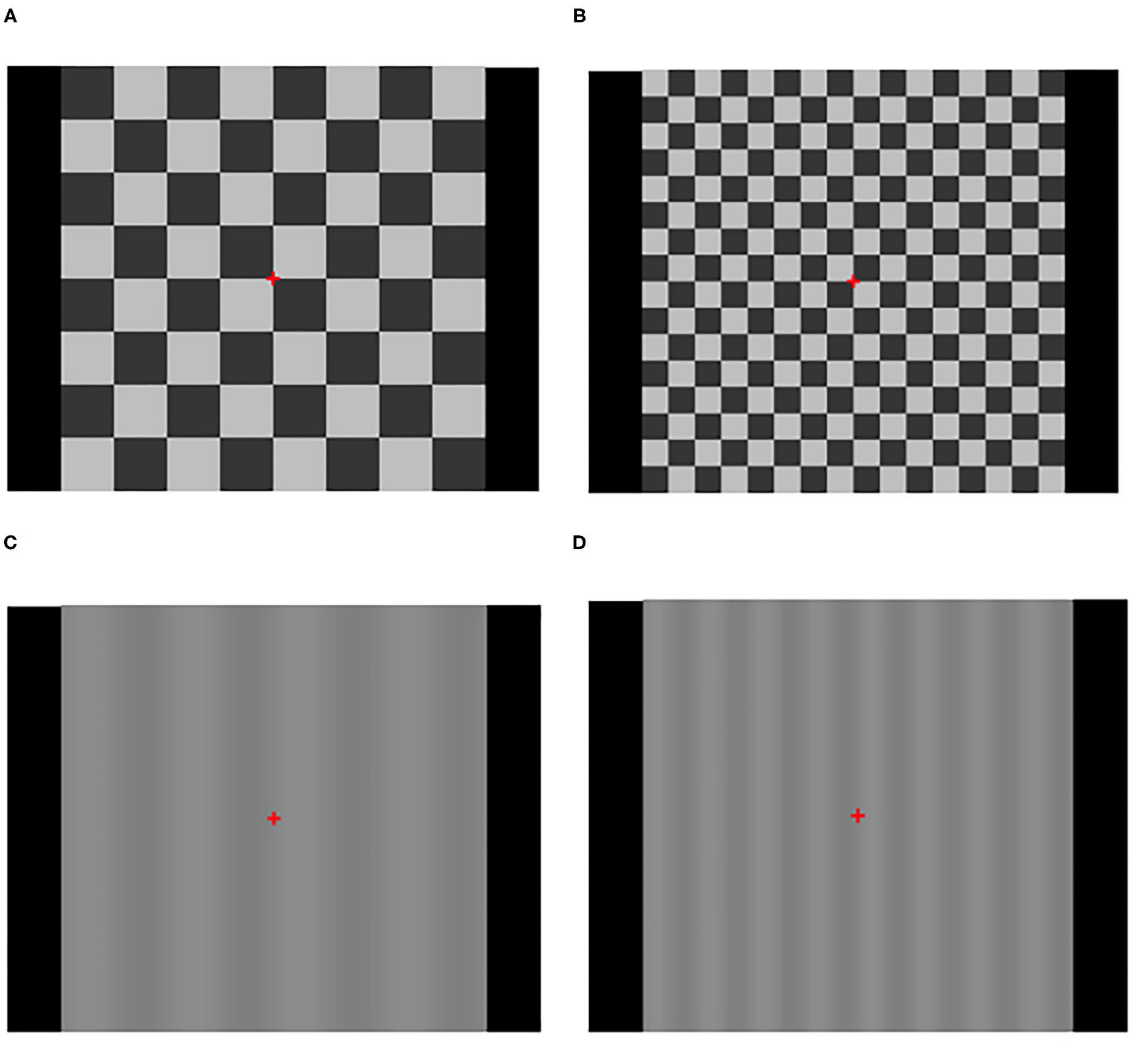
VEP stimuli using checkerboard patterns at 85% contrast and vertical sinusoidal gratings (VSG) at 10% contrast. **(A)** 8 × 8 checkerboard (119.4 MOA checks). **(B)** 16 × 16 checkerboard (59.7 MOA checks). **(C)** 8 VSG (119.4 MOA-wide columns). **(D)** 16 VSG (59.7 MOA-wide columns).

The transient pathway stimuli consisted of 0.25 and 0.50 cycle/deg vertical sine wave gratings pattern reversed at a rate of 4 rev/sec. Both gratings had a Michelson contrast of 10% and mean luminance of 102 cd/m^2^. The 0.25 and 0.50 cycle/deg gratings had a total of 4 and 8 cycles, respectively. Note that the physical width of each half cycle of the 0.25 and 0.50 cycle/deg gratings was equal to the width of the 2 and 1-degree check sizes, respectively (see Figure 1).

In all cases, the display was viewed binocularly through natural pupils with optimal refractive correction in place. The viewing distance was set to 1 meter, yielding a total display viewing angle of 15.92°. During a recording session each stimulus pattern was presented 3 times with each presentation lasting 20-s. The total duration of the stimulus pattern sequence was 240-s. The sequence was performed as follows: 2-degree checkerboard (three times); 0.25 cycle/deg vertical sinusoidal grating (three times); 1 degree checkerboard (three times); 0.5 cycles/deg vertical sinusoidal grating (three times).

Analog signals were amplified by a factor of 20000 (Diopsys Nova Amp, Diopsys, Inc., Pine Brook, New Jersey, USA), band-pass filtered with cut-off frequencies of 0.5–100 Hz and sampled at 1,024 Hz for the checkerboard pattern (512 data points) and 2,048 Hz for the vertical sine patterns (1,024 data points).

The module automatically measured signal-averaged latency of the exogenous P100 component of the typical N75-P100-N135 complex in response to visual stimulus presentation. This latency represents conduction time between retinal stimulation and excitation of neurons in the primary visual cortex. In the context of the present study and consistent with the manufacturer's nomenclature the term “latency” refers to time to the first major positive peak occurring around 100 ms (P100). The module also provided relative amplitude measurements in the form of the difference between the N75 and P100 (delta N75-P100), which is thought to address issues of individual variability attributed to anatomical differences and electrical properties of the testing environment. VEP extraction for this system was previously described by Tello et al. (2010) and is based on the method developed by Derr et al. (2002).

#### Training Instruments

##### VTS4

One of the commercially available clinical devices/software is the VTS4, that is, Vision Therapy System version 4 (HTS Inc., Gold Canyon, AZ, USA). VTS4 is commonly used in optometric/ophthalmologic practices that specialize in binocular vision. VTS4 is used for both diagnosing (e.g., vergence anomalies) and for managing binocular vision anomalies (e.g., eliminating amblyopia; breaking suppressions; improving oculomotor skills; improving visual memory; improving accommodative facility; altering retinal correspondence; increasing fusional ranges; and/or treating strabismus). VTS4/Computer Orthoptics includes complex monocular and binocular stimuli, which allow automatic testing and measurement of the following skills: Oculomotor (pursuits and saccades); fusional vergence ranges; heterophorias; motor fields; fixation disparities, suppressions; retinal correspondence; accommodative facility; stereopsis, visual memory and Aniseikonia. VTS4 uses liquid crystal glasses with dichoptic stimuli that are synchronized to each eye through rapid alternating occlusion to help train the patient. Included, are several random dot stereograms and other stereo targets which are devoid of monocular cues and can be seen only during binocular vision, thus ensuring patient compliance. Specific therapy procedures are designed to improve smooth (ramp) vergences, jump (step) vergences, positive and negative accommodation, pursuits, saccades, sensory fusion, and visual memory.

##### The Neurotracker Training System

The Neurotracker Training system was developed by CogniSens Inc. in collaboration with the National Hockey League and US NCAA to train visual perception span, split attention and decision-making. The program presents randomly moving spheres in 3D space at variable speeds. Four spheres are targeted for tracking and then blend with another four. Repeated trials following a staircase procedure allows athletes to both expand the amount of movement information they can absorb in the field and process that information more efficiently until a training speed threshold is established. As a result of this training, the athlete is able to decrease their anticipatory response time in terms of reading the play, make quicker decisions during play action and increase the time available to choose the best play option (Faubert and Sidebottom, 2013). In their review Appelbaum and Erickson (2018) noted that NeuroTracker performance had been correlated with actual game performance in professional basketball players and university-level soccer players.

##### Nike Vapor Strobe® Glasses

The Nike Vapor Strobe (R) eyewear uses battery-powered liquid crystal filtered lenses that alternate between transparent and opaque states and provide varying lengths of occlusion that are under the control of the participant. The transparent state consists of complete visibility while the opaque state consists of a medium gray that is difficult to see through. The strobe effect is defined by opaque states that can be changed through eight different durations, ranging from 25 to 900 ms of visual occlusion, while the transparent state is fixed at a constant 100 ms (Appelbaum et al., 2011). This means that the amount of time the participant can see through the glasses never changes, but instead, the duration of time the glasses are opaque changes depending on the difficulty level. The shorter the opaque state, the greater the total opportunity to see a moving object. In their 2018 review Applebaum and Erickson suggested that stroboscopic training may be particularly useful for improving dynamic visual acuity, as well as some sport-specific motor skills affecting athletic performance.

##### Sanet Vision Integrator (SVI)

The Sanet Vision Integrator (SVI), developed by Robert B. Sanet and Rodney K. Bortel is a training software available as a computerized system through HTS, INC. It is widely used in clinical optometry and optometric vision therapy to evaluate visual guidance of motor performance and offers five primary programs used for vision training. These programs include: Eye Hand, Rotator, Metronome, Saccades, and Tachistoscope. For the purposes of the present study we used the Rotator, the Saccade and the Tachistoscope modules.

The Rotator module combined 4 rotator programs that work on various aspects of visual ability including Eye-Hand Coordination, Visual Reaction Time, Hand Speed and Accuracy, Peripheral Awareness, Central-Peripheral Integration, Bilateral Integration, Pursuits, Saccades, Visual Acuity Enhancement, Anti Suppression-Monocular Fixation in a Binocular Field (MFBF), Contrast Sensitivity, Vision Directing Action, Visual-Auditory Integration and Sequencing. For the purposes of the present study we used Rotator 1, 2, and 4 programs. In Rotator I targets remain on a virtually rotating screen until the stimulus is touched. The size, color, and shape of the stimulus as well as the speed of the virtual screen rotation can be manipulated within this level. In Rotator II visual target classification and sequencing are added. At this level rotating targets are of different colors and the task is not only to quickly and accurately touch specific targets but to do so in the proper order (e.g., red-white-blue). In Rotator IV the stimuli are upper- and lowercase letters and numbers. These targets need to be hit in a specific order (e.g., alphabetically or in an ascending order, vs, in a reverse alphabetic of descending order).

The saccade module was used for saccading training and included Saccade 1, Saccade 2, and Verbal 1 programs. With Saccade 1, the subject stands in front of a 46-inch touch-screen where various non-duplicated numbers or letters are scattered across the screen. The subject is to rapidly search the screen and touch each number or letter in sequence until all the numbers/letters are gone. In Saccade 2, a single number, letter or word is briefly flashed at a random position somewhere on the screen. The subject must make a foveal saccade to locate the target and accurately call out the target that was flashed. When words are used, there are 200 common sight words that can be automatically flashed to further aid in word coding. The verbal mode is similar to the Saccade 1 program. A large number of numbers or letters are scattered across the screen. The computer calls out the number/letter to be touched and the subject tries to find and touch each number as it is called out by the computer until all the numbers/letters on the screen are gone.

Finally, the Tachistoscope module is designed to train the following visual abilities: Speed and Span of Recognition, Visual Memory, Auditory memory, Auditory-Visual Integration, Visualization, Visual Search, Saccades, Visual Cognitive, Divided Attention. In this module various target stimuli can be used such as letters, numbers and words. The variable stimulus sequence is briefly presented centrally and then disappears, after which elements of the sequence are randomly presented in both central and peripheral screen locations. The goal is to touch the stimulus target in the right sequence (or reverse sequence from the standard).

##### The Quick Board

The Quick Board is a visual motor training technology intended to improve agility and balance. Quick Board's methodology was designed to improve and restore characteristics that are critical to athletic performance by enhancing motor learning with real-time feedback during exercises. It consists of a 41” × 31” × 1 HD Sensor Board with 5 circular targets and an iPad connected to the board. Quick Board's software allows athletic trainers and therapists to customize training protocols based on the subject population (e.g., athletic vs. clinical). Exercises and protocols provided in the software target reaction, speed, quickness, stability, coordination, balance, and proprioception. The device can also administer Go/No-Go type of tasks when sensor targets appear in different colors requiring differential motor output from the trainee. During the exercises, Quick Board's software displays real-time feedback on the iPad so that the subjects train with their eyes focused on the iPad, not looking down at the sensor board. Galpin et al. (2008) reported that training with the Quick Board was associated with significant improvements in foot speed, choice reaction, and change-of-direction in moderately active adults.

##### The Fitlight

The Fitlight is a speed and cognitive training system that is designed to be customizable for sport-specific training. It consists of several (*n* = 8 in the present study) wireless, RGB LED powered lights that are used as targets for the user to deactivate as per the reaction training routine. These training lights can be mounted to walls, poles, and other training equipment or they can be strategically placed on the ground for specific training routines. Each light is controlled via an app. The app can be installed on any hand-held Android or Apple device and is available through the Google Play and App Store. During setup, users can program the lights using one of the built-in programs. Users can also modify programs or create unique regimens themselves. Once the user has selected their desired program, they can begin using the lights. During any type of training, specifically, speed and agility training, the lights can be deactivated by use of the users' hands, feet, head, or sport/fitness/healthcare related equipment. The deactivation of the lights can be achieved through full contact or proximity–waving, running past, swiping, etc. Captured performance data (e.g., hits/misses, average reaction time, total time to hit all targets) are available for each individual athlete in a time series (over multiple training session) and can be used for subsequent group analysis. This system has been recently reported to produce improved neuromuscular control in Russian University-level basketball players characterized by a 43.6% reduction in ball control errors during dribbling (Rogozhnikov et al., 2020).

### Procedure

#### Testing

Prior to enrollment into the study, informed consent was obtained from both parents/ legal guardians of youth between 13 and 18 as well as participating minors. Participants under 16 were also given a simplified assent form, in which the study and their participation was explained in plain every-day terms.

All testing was conducted on the premises of a local optometric clinic. All enrolled participants underwent a standard optometric exam administered by a licensed optometrist. The optometric tests that we performed on the athletes included distance visual acuity (Snellen Chart), near visual acuity (Reduced Snellen Chart) and non-cycloplegic manifest refraction. If significant refractive error was discovered (≥+1.00 sph or −0.50 sph or ≥-0.75 cylinder) the subject was fitted with MyDay daily disposable contact lenses (Cooper Vision) to achieve at least 20/20 vision in each eye. Through these lenses we measured: distance phoria (Von Graefe); near phoria (modified Thorington); nearpoint of convergence (NPC using Bernell Rule); monocular accommodative pushup amplitudes (Bernell Rule); vergence facility at near (3BI/12BO prism); accommodative facility (+2.00/−2.00 flippers) phoropter-based near testing including negative relative vergence; positive relative vergence; positive relative accommodation; negative relative accommodation; and phoropter-based associative vergence measures and Nearpoint of Fixation Disparity (NPFD by Vision Assessment Corporation). The full description of oculomotor protocols used in the present study can be found in Poltavski and Biberdorf (2014).

After the optometric evaluation participants underwent VEP testing. The VEP recording utilized a 3-electrode montage using Diopsys skin electrodes. The active electrode was placed ~4 cm above the inion in the Oz location according to the International 10–20 system (i.e., primary visual cortex) while the reference electrode was placed about 10–11 cm above the nasion (Fz location, according to the International 10–20 system). The left side of the forehead (position Fp1, according to 10–20 system) served as ground. In preparation for recording, the skin at each electrode site was scrubbed with Nuprep (D.O. Weaver & Co., Aurora, CO) on a cotton-tipped wooden swab. Electrodes were fixed in position with Ten20 conductive paste (D.O. Weaver & Co., Aurora, CO) and secured with a small gauze pad with conductive paste applied. Electrode impedance was maintained below 10 k ohms in all cases and was usually below 5 k ohms.

Each subject was instructed to sit comfortably and steadily about 1 meter from the test screen and centered along the midline at eye level and blink normally during the procedure. Per the manufacturer's software, a small (0.25° radius) red rotating, annular fixation cross target was presented in the center of the test screen to control accuracy of fixation and accommodation as well as to maintain visual attention. Subjects were instructed to fixate upon the small central target with minimal blinking to reduce any response artifacts. Three 20-s trials were conducted for each stimulus type (checkerboard vs. vertical sinusoidal grating) and size (0.25 cycle/deg vs. 0.50 cycle/deg). The participant had to complete all 3 presentations of the same pattern before proceeding to the next one. Trials were separated by ~3-s breaks. During the breaks the experimenter encouraged the participant to continue looking at the center of the screen at the fixation target and manually initiated the next trial. Each pattern was thus presented for 1 min resulting in the total of 4 min of VEP recording.

All Nike SST/Senaptec and EEG testing sessions were completed after the optometric and VEP evaluation and on a separate day. Upon arrival to the lab, each participant was fitted with the EEG sensor strip that was plugged into the B-Alert X-10 wireless sensor headset. Once all channel impedance values were below the manufacturer recommended 40 kΩ, each participant first underwent a 15 min neuropsychological evaluation consisting of three 5 min computerized tasks programmed by the manufacturer of the acquisition software (ABM) to automatically generate cognitive state metrics (these statistics were not utilized in this study).

Following completion of the benchmark tests, participants underwent the Nike SST/Senaptec assessment. Each station test was preceded by a brief practice session that included several trials. Within the B-Alert X-10 Live Acquisition software, the continuous EEG stream was broken into intervals corresponding to the beginning and end of each Nike SST task by manually inserting start and end markers during acquisition. Once the participant completed all the tasks, acquisition of EEG signals was terminated, the headset and the sensor strip were removed, the participant was debriefed and dismissed. Each assessment session lasted on average 60–65 min.

#### Training

The subjects were randomly assigned to one of the two training protocols. Both protocols involved two 1 h training sessions per week for 10 weeks resulting in a total of 20 h of combined training per subject. Group one (hardware-software group) completed 5 weeks (10 h) of oculomotor training first followed by 5 weeks (10 h) of software training. For group 2 (software-hardware) the order of procedures were reversed. All training procedures were administered by the same Optometric Vision Therapist (OVT)/SVT trainer who was also Nike-certified in Sensory station training and testing.

Visual hardware training was based on the general principles and guidelines for office-based vision therapy (VT)/Orthoptics developed by the Convergence Insufficiency Treatment Trial (CITT) Study Group (2008). This program was divided into 3 phases. Within each phase there were a number of endpoints within the techniques for each category such as gross convergence, vergence, and accommodation. The therapy procedures in each category were arranged sequentially from easiest to most difficult. Phase I focused on building monocular oculomotor and accommodative skills, stabilizing the vergence system at far and near and improving gross convergence. Subjects of average spent 2 to 3 h of their entire training in Phase I of the hardware training. The objective of phase II was to improve binocular integration focusing on binocular accommodative and oculomotor tasks by having the subject make smooth (ramp) vergences to changing demands and integrating vergence and accommodation for increased accuracy. Similarly to Phase I, the participants spent between two and three 1 h sessions practicing drills in Phase II. The emphasis of Phase III was to build visual automaticity to habituate the newly learned skills. Therapy in this phase challenged the subject to increase the speed of binocular jump vergences and accommodation to improve their facility. Up to 3 h was spent practicing activities in Phase III. The 10th hour of hardware training (i.e., final session) was dedicated to reviewing oculomotor skills developed during the three phases of training and included fusion, accommodation, saccadic eye movements, tracking, visual tracing, and suppression.

The following techniques and activities were used to accomplish these goals in different phases. Brock String and Barrel Cards were used in Phase I for gross convergence training. Vectograms (Quoits/Clown) Base Out, Computer Othoptics (VTS4: Random Dot Stereograms, Jump Ductions, Visual memory, Saccades) and Life Saver Cards were used in Phase 1 for fusional vergence training. Loose Lens Accommodative Rock, and Letter Chard Accommodative Rock were used in Phase I for accommodative training. In Phase II Fusional Vergence training was further developed with Vectograms (Quoits/Clown), Computer Orthoptics (VTS4: RDS, Jump Ductions, Visual memory, Saccades), Aperture Rule and Eccentric Circles. Accommodative training in Phase II was implanted using the Loose Lens Accommodative Rock, Accommodative Flippers and the Letter Chart Accommodative Rock. In Phase III jump vergence procedures were used for fusional vergence training with the following techniques: Vectrograms (Quoits/Clown), Computer Orthoptics (VTS4), Aperture Rule, Eccentric Circles and Loose Prism Facility. Accommodative training goals in Phase III were accomplished using Binocular Accommodative Flippers. Detailed procedures for administration of these techniques are described in the Convergence Insufficiency Treatment Trial Manual of Procedures (https://optometry.osu.edu/CITT-manual-procedures).

For visual software training at every training session each subject completed 2 rounds of the following activities: four original Nike training modules (Eye-Hand Coordination, Go/No-Go, Depth Perception and Split Attention), the Quick Board, the Fitlight and the Neurotracker Core Training Session, slide board and rubber board drills. The Nike Vapor Strobe glasses were used during the Eye-Hand Coordination and Go/No-Go training sessions at different Levels starting from Level 1 up to Level 8 and were added to the training following week 1 (after 2 h of training on the four modules). When the strobe glasses were used the Eye-Hand Coordination and the Go/No-Go modules were be completed twice: once without the glasses and once with the strobe glasses on. These Nike activities were practiced during all software training sessions with the difference in the speed of stimulus presentation and the Level of the Nike Vapor Strobe glasses. After the first 2 weeks of training (after 4 h) the challenge level of these activities was further increased by placing participants on the balance ball.

Following completion of the Nike training module activities, participants proceeded to the Quick Board, which was also used in combination with the Nike Vapor Strobe Glasses (added to the regimen after week 1) and a hockey stick with a padded blade to make visual drills more sport-specific. Various sequences of lit-up targets on the board had to be touched both with the left and right feet as well as the hockey stick with and without Strobe Glasses. After the Quick Board activities, the participants moved on to the Fitlight training. A random setup sequence of 8 Fitlights arranged on the floor was used in hockey-specific training drills when the study participants were asked to use a hockey stick with the padded blade to hit each randomly illuminated target once with the Nike Vapor Strobe glasses on and once without the glasses.

After the Fitlight drills, the participants completed 8 min of the Neurotracker Core Training Session with variable speeds, which depended on the subject's accuracy during a preceding trial (increased speed if the subject was correct, and decreased speed, if the subject was incorrect). Then the participant was placed on a sliding board and would start sliding side to side while the therapist/trainer (positioned 10 feet away) would throw a tennis ball at the subject to the side to which the subject was moving. The objective of the activity was to catch the ball and to throw it back to the trainer while sliding. The activity took 2 min to complete and was intended to mimic eye-hand coordination demands while ice-skating. During the second minute Nike Vapor Strobe Glasses were added to the activity set at Level 1 or 2. The final activity of round 1 included practicing catching a rubber ball on an elastic string affixed to the wrist of the throwing hand of the subject for 4 min. The objective would be to bounce the ball off the floor and catch it. Each subject would switch hands after 1 min. Following the first 2 min the Nike Vapor Strobe Glasses set at level 1 or 2 were added to the drill. After these activities were completed the subject would repeat the cycle (Round 2). Completion of round 2 signified the end of the training session. It took on average 1 h to complete all of the activities in rounds 1 and 2.

#### Statistical Analyses

For assessment of performance changes on the Sensory Station measures as a function of training (time) and training protocol type (treatment) we combined data from equivalent measures of the Nike SPARQ Sensory System and the Senaptec Sensory station and included it into overall analyses. The within-subject variability was maintained for each station, so that error variance attributable to potential discrepancies in measurements between the two systems was not part of within-subject variance (i.e., each subject was tested all 3 times either on one or the other system).

Our 2 treatment groups did not significantly differ on any of the baseline measures of the Nike/Senaptec Sensory Stations, STMLI ratios or VEP (see Table 1 for details). There were no group differences in the participant age. We, therefore, did not include baseline measurement as a co-variate in any of the analyses and used it only as part of an outcome variable (i.e., testing differences between the baseline values and follow-up measurements).

**Table 1:** Means and standard deviations for dependent variables at baseline as a function of treatment order.

**Variable Name**	**Treatment Order**				**t (t_**crit**_ at α = 0.05 and 50 df = 2.01)**
	**Hardware-Software (*n = * 22)**		**Software-Hardware (*n =* 31)**		
	**Mean**	**SD**	**Mean**	**SD**	
**Age**	14.27	1.20	13.58	1.28	0.06
**Nike/Senaptec**					
Visual Acuity (logMAR)	−0.11	0.13	−0.10	0.17	−0.48
Contrast Sensitivity (logCS): 6 cpd	2.12	0.19	2.10	0.20	0.82
Contrast Sensitivity (logCS): 18 cpd	1.49	0.35	1.43	0.28	0.59
Depth Perception (arcsec)	127.18	99.65	91.65	88.89	1.90
Near-Far Quickness	21.74	4.52	22.09	4.26	−0.57
Target Capture (ms)	284.68	158.73	297.73	146.49	0.36
Eye-hand Coordination (ms)	58,773.27	6,740.64	58,675.71	5,890.32	0.06
Go/No-Go	8.86	6.71	9.94	6.80	−0.0.57
Hand Reaction Time (ms)	385.75	39.92	391.94	50.59	−0.48
Perception Span	38.95	15.12	36.74	12.40	0.58
**Short-Term Memory Load Index (STMLI**) **for Nike/Senaptec tests (μV**^**2**^**)**					
Visual Acuity	0.47	0.07	0.52	0.13	−1.53
Contrast Sensitivity	0.45	0.07	0.50	0.13	−1.62
Depth Perception	0.55	0.09	0.60	0.10	−1.82
Near-Far Quickness	0.46	0.08	0.50	0.13	−1.51
Target Capture	0.50	0.09	0.51	0.12	−0.63
Eye-hand Coordination	0.54	0.09	0.60	0.13	−1.78
Go/No-Go	0.54	0.10	0.59	0.13	−1.47
Hand Reaction Time	0.54	0.12	0.53	0.11	0.16
Perception Span	0.50	0.11	0.53	0.13	−0.90
**VEP Latency (ms)**					
8 × 8 Checkerboard Mean	103.99	7.82	106.43	6.31	−1.23
8 Vertical Sinusoidal Mean	116.05	13.52	114.94	12.62	0.30
16 × 16 Checkerboard Mean	105.87	7.97	104.81	5.11	0.59
16 Vertical Sinusoidal Mean	122.73	13.47	119.78	18.77	0.62
**VEP Amplitude (μV)**					
8 × 8 Checkerboard Mean	18.59	4.56	20.53	5.39	−1.37
8 Vertical Sinusoidal Mean	11.56	3.30	13.69	4.43	−1.25
16 × 16 Checkerboard Mean	18.84	5.86	20.44	6.30	−0.93
16 Vertical Sinusoidal Mean	11.72	3.94	13.02	3.50	−1.86

Based on the previously identified dimensions for the Nike Sensory station (Wang et al., 2015), we conducted three doubly multivariate Analyses of Variance (Repeated Measures MANOVAs) separately for measures of visuo-motor control, visual sensitivity and Eye Quickness. We grouped STMLI measures collected during these tasks similarly and conducted similar doubly multivariate analyses of variance with time of assessment (baseline, after 5 weeks of training and after 10 weeks of training) being the within-subject variable and treatment order (hardware—software or software-hardware)—the between-subject variable. Univariate assumptions of normality were tested using histograms, Kolmogorov-Smirnov and Shapiro-Wilks tests as well as skewness statistics exceeding 2 standard errors. Screening for univariate outliers was conducted by examining standardized z-scores on individual dependent variables exceeding 3.29 (α = 0.001). Multivariate outliers were identified using Mahalanobis distances exceeding critical χ^2^ values with df equal to the number of observations at α = 0.001.

VEP data was collected in the form of latencies (ms) and amplitudes (μV) of the N75-P100-N135 complex (henceforth referred to as P100) in response to the two checkerboard and two vertical sinusoidal grating stimuli. Each stimulus was associated with 3 outcome response measures of latency and amplitude: fastest response to the type and size of the stimulus, medium response and slowest response. We averaged response latencies and amplitudes over the three responses and used the resultant means as dependent measures. Our preliminary diagnostics showed significant deviations from normality for most latency and amplitude variables based on significant Kolmogorov-Smirnov and Shapiro-Wilks tests as well as positive skewness statistics exceeding 2 standard errors for most variables. Thus, prior to statistical analyses all VEP variables underwent log10 transformation to minimize issues with normality and univariate outliers. This type of transformation is recommended for substantial issues with normality as identified by the shape of the distribution and corresponding z-scores of deviation statistics of skewness and kurtosis >3.29 (Tabachnick and Fidell, 2019).

We conducted doubly multivariate analyses of variance (Repeated Measures MANOVAs) separately for four log10-transformed latency variables (log10 mean latency for 16 × 16 checkerboard pattern, log10 mean latency for 8 × 8 checkerboard pattern, log10 mean latency for a 16-column vertical sinusoidal grating and log10 mean latency for an 8-column vertical sinusoidal grating) and corresponding 4 log10-transformed amplitude variables. Time of assessment (baseline, after 5 weeks of training and after 10 weeks of training) was the within-subject variable and treatment order (hardware—software or software-hardware) was the between-subject variable. Normality of the distributions for the log10-transformed variables was verified by comparing z-score values for skewness and kurtosis against the critical value of 1.96 at alpha level of 0.05.

Power calculations were based on follow-up univariate Repeated Measures Analyses of Variance looking for interaction effects for within-and-between subject variables on any given dependent measure. Using G-Power 3.1 (Faul et al., 2009) we estimated that with 53 participants, 2 between-subject groups and 3 repeated measurements we could detect small-to-medium effect sizes (f = 0.17) while maintaining minimal acceptable statistical power of 0.80, alpha level of 0.05 while assuming a conservative correlation between any two repeated measurements of 0.5.

## Results

### Nike/SENAPTEC Sensory Station

#### Measures of Visual Sensitivity

A significant Box's M test (F = 1.33 *p* = 0.02) indicated a violation of the homoscedasticity assumption for dependent variables. Therefore, multivariate significance was assessed by evaluating significance levels associated with the F statistic for Pillai-Bartlett Trace test as a more robust measure to the violation of the homogeneity of covariances assumption. The results indicated multivariate significance for the main effect of time (Pillai's Trace F = 7.07, *p* < 0.01). This multivariate effect was associated with sufficient statistical power (1.00) and a moderate effect size (partial η^2^ = 0.49). Neither the effect of treatment order, nor the order × time interaction were significant (see Table 2).

**Table 2:** Means, Standard Deviations, and Two-way ANOVA statistics for Measures of Visual Sensitivity.

**Variable**	**Hardware-Software (*n =* 22)**		**Software Hardware (*n =* 31)**		**Total (*N =* 53)**		**Effect**	**ANOVA**		
	**M**	**SD**	**M**	**SD**	**M**	**SD**		**F**	**η^2^**	**Power**
**Visual Acuity (logMAR)**										
Baseline	−0.11	0.13	−0.10	0.17	−0.10	0.15	T	4.55^*^	0.07	0.77
5 Weeks	−0.15	0.10	−0.15	0.15	−0.15	0.13	G	0.01	0.00	0.05
10 weeks	−0.15	0.10	−0.17	0.09	−0.16^a^	0.10	T X G	0.25	0.00	0.09
**Contrast Sensitivity (logCS): 6 cpd**										
Baseline	2.12	0.19	2.10	0.20	2.11	0.19	T	0.36	0.00	0.11
5 Weeks	2.13	0.17	2.13	0.16	2.13	0.17	G	0.02	0.00	0.05
10 weeks	2.09	0.25	2.12	0.25	2.10	0.25	T X G	0.41	0.01	0.11
**Contrast Sensitivity (logCS): 18 cpd**										
Baseline	1.49	0.35	1.43	0.28	1.46	0.31	T	1.67	0.03	0.35
5 Weeks	1.50	0.24	1.49	0.24	1.49	0.24	G	0.32	0.01	0.08
10 weeks	1.55	0.32	1.52	0.31	1.53	0.31	T X G	0.21	0.00	0.09
**Depth Perception (arcsec)**										
Baseline	127.18	99.65	91.65	88.89	109.15	95.32	T	17.80^**^	0.22	1.00
5 Weeks	71.09	69.77	68.71	69.54	69.88^a^	69.14	G	3.01	0.04	0.40
10 weeks	55.55	57.18	30.91	21.23	43.04^a^^,^ ^b^	44.33	T X G	1.15	0.02	0.25

*G, group; T, time*.

* *p <0.05*.

** *p <0.01*;

a *significantly different from baseline at α = 0.05*;

b *significantly different from 5 week assessment at α = 0.05*.

Univariate analyses showed that the multivariate significance for the main effect of time was significant for visual acuity and depth perception but neither of the two variables of contrast sensitivity. Mauchly's test of sphericity was not significant for visual acuity (*p* = 0.16), so sphericity was assumed. The significant main effect of time [*F*_(2, 130)_ = 4.55, *p* = 0.01] was associated with a small effect size (η^2^ = 0.07) and sufficient power (power = 0.77). Pairwise comparisons with the Sidak adjustment showed a significant progressive decrease in the Logarithm of the Minimum Angle of Resolution (logMAR) that reached statistical significance at alpha = 0.05 between baseline (M = −0.10, SE = 0.02) and final assessment (M = −0.16; SE = 0.01) indicating improved visual acuity.

Mauchly's test of sphericity was significant for depth perception (*p* < 0.01) indicating a significant violation of the sphericity assumption (χ^2^ = 21.22, *p* < 0.01) for that variable and requiring an epsilon (ε) adjustment of the degrees of freedom for the corresponding F-test. Since the Greenhouse-Geisser ε was >0.7 for depth perception, the Huyhn-Feld ε-adjusted F statistic was used to assess significance of the univariate test [*F*_(2, 130)_ = 17.98, *p* < 0.01, power = 0.99, partial η^2^ = 0.22]. Pairwise comparisons with the Sidak adjustment showed a significant progressive improvement in depth perception as indicated by a decrease in the threshold (arc sec) necessary to achieve stereopsis. These differences were significant between baseline and (M = 109.41, SE = 11.53) and the mid-point assessment (M = 69.89, SE = 8.51) and between the 5 week assessment and the 10 week evaluation (M = 43.23, SE = 5.24). These results are summarized in Table 2.

#### Measures of Eye Quickness

The results indicated multivariate significance for the main effect of time [Wilks' Lambda *F*_(2, 59)_ = 9.88, *p* < 0.01]. This multivariate effect was associated with sufficient statistical power (1.00) and a moderate effect size (partial η^2^ = 0.40). Neither the effect of treatment order, nor the order × time interaction were significant.

Univariate analyses showed that the multivariate significance for the main effect of time was significant for both measures of near-far quickness and dynamic visual acuity (target capture). Mauchly's test of sphericity was not significant for either dependent variable, so sphericity was assumed. The significant main effect of time [*F*_(2, 124)_ = 19.11, *p* < 0.01] for near-far quickness was associated with a small effect size (η^2^ = 0.24) and sufficient power (power = 1.00). Pairwise comparisons with the Sidak adjustment showed a significant progressive improvement in the mean number of correctly identified targets between baseline (M = 21.92, SE = 0.56) and the 5 week assessment (M = 24.29, SE = 0.66) and between the 5 week assessment and the final evaluation (M = 26.39; SE = 0.65) (see Table 3).

**Table 3:** Means, Standard Deviations, and Two-way ANOVA statistics for Eye Quickness Variables.

**Variable**	**Hardware-Software (*n =* 22)**		**Software-Hardware (*n =* 31)**		**Total (*N =* 53)**		**Effect**	**ANOVA**		
	**M**	**SD**	**M**	**SD**	**M**	**SD**		**F**	**η^2^**	**Power**
**Near-Far Quickness**										
Baseline	21.74	4.52	22.09	4.26	21.92	4.44	T	19.11^**^	0.24	1.00
5 Weeks	25.45	4.09	23.12	6.16	24.25	5.35	G	1.06	0.02	0.17
10 weeks	26.84	5.11	25.94	5.35	26.38	5.21	T X G	1.72	0.03	0.35
**Target Capture (ms)**										
Baseline	284.68	158.73	297.73	146.49	291.41	151.46	T	4.84^*^	0.07	0.80
5 Weeks	255.65	110.06	270.45	130.12	263.28	120.10	G	0.14	0.00	0.07
10 weeks	229.03	96.19	223.48	98.02	226.17	96.41	T X G	0.15	0.00	0.07

*G, group; T, time*.

* *p <0.05*.

** *p <0.01*;

*^a^significantly different from baseline at α = 0.05*;

*^b^ significantly different from 5 week assessment at α = 0.05*.

The significant main effect of time for dynamic visual acuity [*F*_(2, 130)_ = 4.84, *p* = 0.01] was associated with adequate power (power = 0.79) and a small effect size (partial η^2^= 0.08). Pairwise comparisons with the Sidak adjustment showed a significant progressive improvement in the time (ms) required to correctly identify the direction of the opening in the Landolt ring. These differences were significant between baseline and (M = 291.20, SE = 19.08) and the final assessment (M = 226.26, SE = 12.14). These results are summarized in Table 3.

#### Measures of Visuo-Motor Control

The results indicated multivariate significance for the main effect of time (Pillai's Trace F = 33.67, *p* < 0.01). This multivariate effect was associated with sufficient statistical power (1.00) and a large effect size (partial η^2^= 0.86). Neither the effect of treatment order, nor the order × time interaction were significant.

Univariate analyses revealed that the multivariate significance for the main effect of time was significant for all of the individual dependent variables. Mauchly's test of sphericity was not significant for all but one DV (i.e., Go/No-Go) indicating a significant violation of the sphericity assumption (χ^2^ = 17.79, *p* < 0.03) for that variable and requiring an epsilon (ε) adjustment of the degrees of freedom for the corresponding F-test. The Huyhn-Feld ε-adjusted F statistic was used to assess significance of the univariate test [*F*_(2, 102)_ = 43.46, *p* < 0.01, power = 1.00, partial η^2^ = 0.46]. Pairwise comparisons with the Sidak adjustment showed a significant progressive increase in the Go/No-Go total score from baseline (M = 9.4, SE = 0.94) to the mid-point assessment (M = 17.58, SE = 1.48) and from the 5 week assessment to the 10 week evaluation (M = 23.53, SE = 1.68). These results are summarized in Table 4.

**Table 4:** Means, Standard Deviations, and Two-way ANOVA statistics for Visuo-Motor Variables.

**Variable**	**Hardware-Software (*n =* 22)**		**Software-Hardware (*n =* 31)**		**Total (*N =* 53)**		**Effect**	**ANOVA**		
	**M**	**SD**	**M**	**SD**	**M**	**SD**		**F**	**η^2^**	**Power**
**Eye-hand Coordination (ms)**										
Baseline	58,773.27	6,740.64	58,675.71	5,890.32	58,716.21	6,194.23	T	82.21^**^	0.62	1.00
5 Weeks	55,814.27	6,968.43	53,669.94	5,880.96	54,560.04^a^	6,379.78	G	0.51	0.01	0.11
10 weeks	50,293.18	4,650.70	49,497.26	4,929.56	49,827.64^a^^,^, ^b^	4,786.56	T X G	1.14	0.02	0.25
**Go/No-Go**										
Baseline	8.86	6.71	9.94	6.80	9.49	6.72	T	43.46^**^	0.46	1.00
5 Weeks	14.05	8.63	21.13	11.78	18.19^a^	11.07	G	2.49	0.05	0.34
10 weeks	22.45	12.20	24.61	11.89	23.72^a^^,^ ^b^	11.95	T X G	2.21	0.04	0.44
**Hand Reaction Time (ms)**										
Baseline	385.75	39.92	391.94	50.59	389.37	46.14	T	10.27^**^	0.17	0.98
5 Weeks	375.58	30.03	380.06	35.87	378.20	33.34	G	0.53	0.01	0.11
10 weeks	362.61	23.95	371.46	36.11	367.79^a^	31.68	T X G	0.11	0.00	0.07
**Perception Span**										
Baseline	38.95	15.12	36.74	12.40	37.66	13.50	T	16.16^**^	0.24	0.99
5 Weeks	46.14	13.44	43.00	11.26	44.30^a^	12.19	G	0.622	0.01	0.12
10 weeks	47.00	13.12	44.65	14.38	45.62^a^	13.79	T X G	0.05	0.00	0.06

*G, group; T, time*.

* *p <0.05*.

** * p <0.01*;

a *significantly different from baseline at α = 0.05*;

b *significantly different from 5 week assessment at α = 0.05*.

A significant univariate main effect of time was observed for Eye-Hand Coordination [*F*_(2, 102)_ = 82.21, *p* < 0.01, power = 1.00, partial η^2^ = 0.62]. Pairwise comparisons with the Sidak adjustment showed a significant progressive decrease in the time required to respond to all 80 targets expressed in milliseconds. EHC time significantly decreased from baseline (M = 58,724.49; SE = 871.78) to the mid-point assessment (M = 54,742.10, SE = 885.28) and between the 5 week assessment and the final 10 week evaluation (M = 49,895.22, SE = 671.37). These results are summarized in Table 4.

A significant univariate main effect of time was observed for Hand Response Time [*F*_(2, 102)_ = 10.28, *p* < 0.01, power = 0.99, partial η^2^ = 0.17]. Pairwise comparisons with the Sidak adjustment showed again a progressive decrease in the average response time to a target stimulus expressed in milliseconds. Hand response time significantly decreased between baseline (M = 388.84, SE = 6.48) and the 10 week assessment (M = 367.04, SE = 4.42). The 5-week assessment HRT showed an intermediate value (M = 377.82, SE = 4.68) that failed to reach significance when compared to baseline (*p* = 0.73) and barely missed significance compared to the final assessment (*p* = 0.52). These results are summarized in Table 4.

A significant univariate main effect of time was observed for Perception Span [*F*_(2, 102)_ = 16.16, *p* < 0.01, power = 0.99, partial η^2^ = 0.24]. Pairwise comparisons with the Sidak adjustment showed a progressive increase in perception span (PS). PS total scores were significantly greater at the 5 week assessment point (M = 44.57, SE = 1.7) and the final 10 week assessment (M = 45.82, SE = 1.93) compared to baseline (M = 37.85, SE = 1.89) but did not differ significantly from each other (*p* = 0.73). These results are summarized in Table 4.

### Short-Term Memory Load Index (STMLI)

#### Measures of Visual Sensitivity

The results indicated multivariate significance for the main effect of time (Wilk's Lambda F = 2.54, *p* = 0.03; power = 0.78; partial η^2^= 0.49). Neither the effect of treatment order, nor the order x time interaction were significant. Univariate analyses showed that the multivariate significance for the main effect of time was significant only for visual acuity [Huynh-Feld *F*_(2, 88)_ = 4.40 *p* < 0.02, power = 0.68, partial η^2^ = 0.09] but not for depth perception or contrast sensitivity. Pairwise comparisons with the Sidak adjustment showed a significant progressive decrease in the STMLI ratios over time that reached statistical significance at alpha = 0.05 between baseline (M = 0.49, SE = 0.02) and final assessment (M = 0.44; SE = 0.01).

#### Measures of Eye Quickness

**Figure 2:**
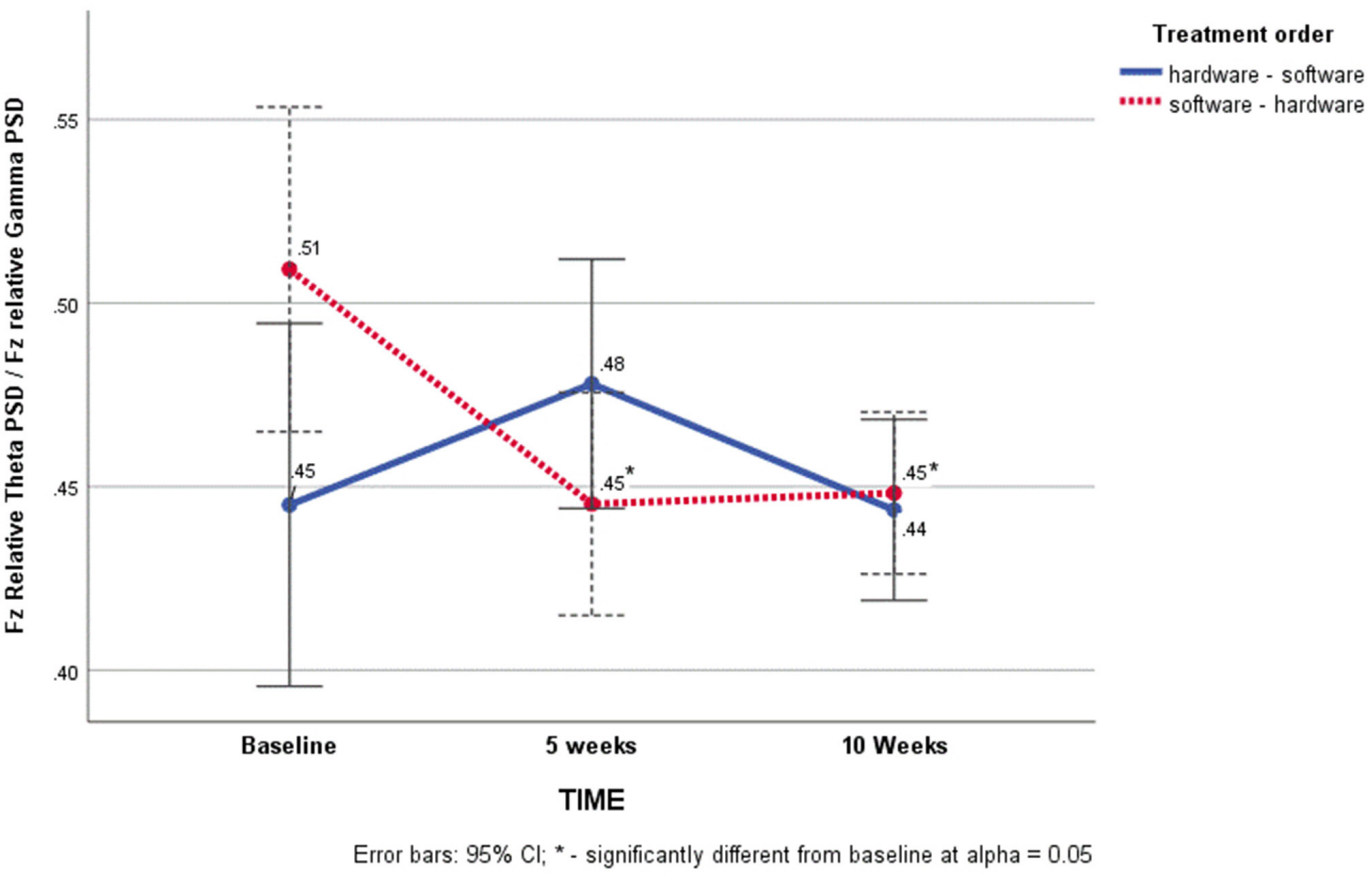
Changes in Short-term Memory Load Index on the Near-Far Quickness Task as a function of time and treatment order.

The results indicated multivariate significance for the time x treatment order interaction [Pillai's Trace *F*_(4, 40)_ = 2.61, *p* = 0.03; power = 0.72; partial η^2^ = 0.06]. Neither the effect of treatment order, nor the effect of time were significant. Univariate analyses showed that the multivariate significance for the time × treatment order interaction was only significant for the measure of near-far quickness [Huynh-Feld *F*_(2, 84)_ = 4.29, *p* = 0.02; power = 0.73; η^2^= 0.09] but not dynamic visual acuity (target capture). This significant time x treatment order interaction was further broken down by simple-effects analyses on near-far quickness within order. A significant main effect of time was observed for the software-hardware training order (*n* = 26, Greenhouse-Geisser F = 4.29, *p* = 0.03). Pairwise comparisons with the Sidak adjustment showed that STMLI ratios were significantly lower than baseline (M = 0.51, SE = 0.03) after both 5 (M = 0.44, SE = 0.01) and 10 weeks of training (M = 0.45, SE = 0.01). There was no significant main effect of time within the hardware-software training order (see Figure 2).

#### Measures of Visuo-Motor Control

**Figure 3:**
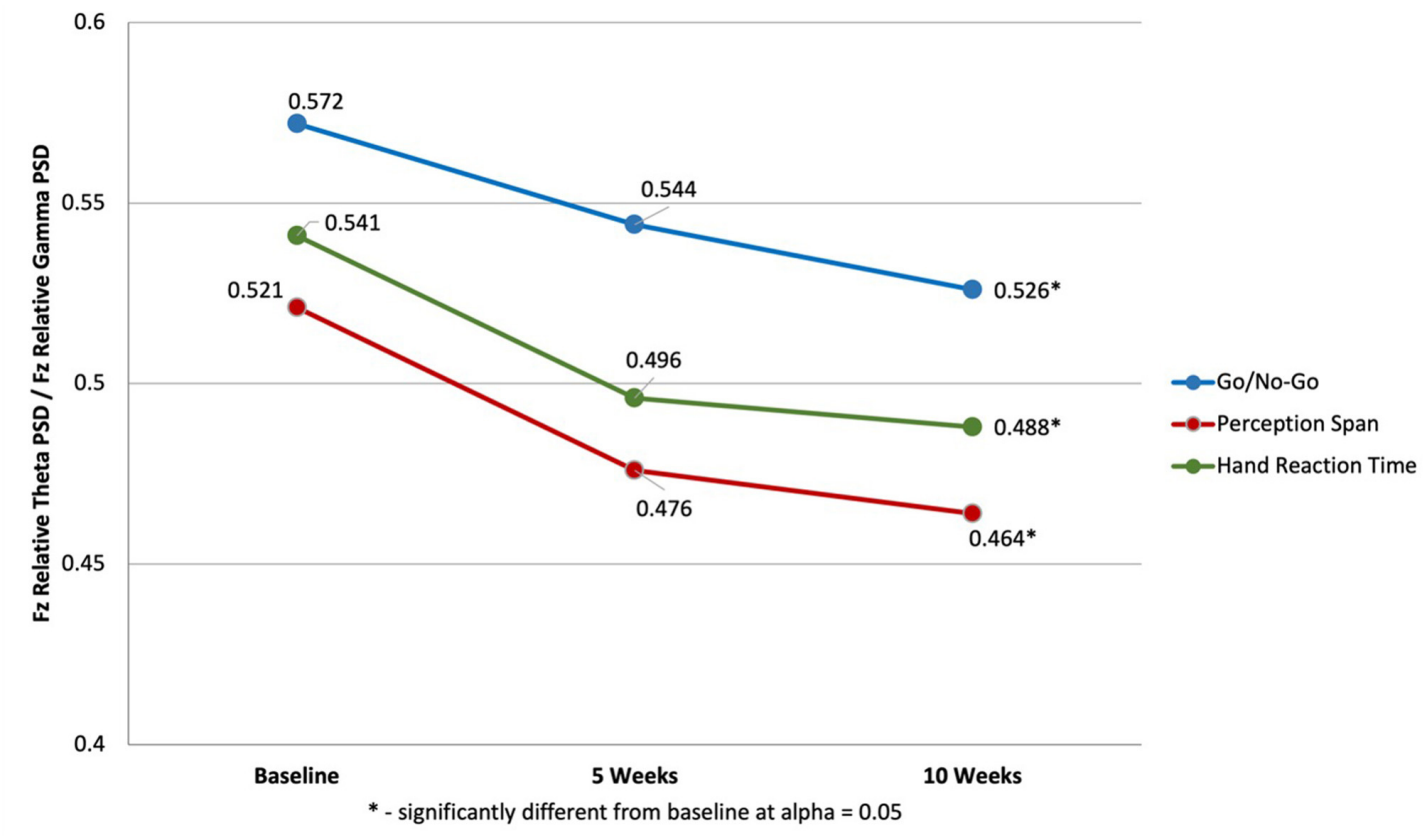
Changes in Short-term Memory Load Index on Visuo-Motor Control measures of Go/No-Go, Perception Span and Hand Reaction Time.

The results indicated multivariate significance for the main effect of time [Roy's gcr *F*_(4, 36)_ = 3.56, *p* = 0.01; power = 0.85; partial η^2^ = 0.15]. Neither the effect of treatment order, nor the order × time interaction were significant. Univariate analyses showed that the multivariate significance for the main effect of time was significant for STMLI ratios on Go/No-Go [*F*_(2, 86)_ = 3.06, *p* = 0.05; power = 0.58; partial η^2^ = 0.07], perception span [Huynh-Feld *F*_(2, 86)_ = 5.21 *p* = 0.01, power = 0.76, partial η^2^ = 0.11] and hand reaction time [Huynh-Feld *F*_(2, 86)_ = 5.66 *p* < 0.01, power = 0.81, partial η^2^ = 0.12] but not for eye-hand coordination. Pairwise comparisons with the Sidak adjustment showed a significant progressive decrease in the STMLI ratios over time that reached statistical significance at alpha = 0.05 between baseline and final assessment on the Go/No-Go test (M_1_= 0.57, SE = 0.02 vs. M_3_ = 0.52; SE = 0.01) perception span (M_1_= 0.52, SE = 0.02 vs. M_3_ = 0.46; SE = 0.01) and hand reaction time (M_1_= 0.54, SE = 0.02 vs. M_3_ = 0.48; SE = 0.01). These results are summarized in Figure 3.

### Visual Evoked Potentials (VEPs)

#### Latencies

A significant Box's M test (F = 1.31, *p* = 0.04) indicated a violation of the homoscedasticity assumption for dependent variables. Therefore, multivariate significance was assessed by evaluating significance levels associated with the F statistic for Pillai-Bartlett Trace test as a more robust measure to the violation of the homogeneity of covariances assumption. The results indicated multivariate significance for the time x treatment order interaction (Pillai's Trace F = 2.27, *p* = 0.044). This multivariate effect was associated with sufficient statistical power (0.80) and a moderate effect size (partial η^2^ = 0.34). Neither the effect of time nor order was significant.

Univariate analyses revealed that the multivariate significance for the time × treatment order interaction was primarily associated with latency differences in response to the 8 × 8 checkerboard pattern. Mauchly's test of sphericity for that dependent variable showed a significant violation of the sphericity assumption (χ^2^ = 7.25, *p* = 0.03) requiring an epsilon (ε) adjustment of the degrees of freedom for the corresponding F-test. Since the Greenhouse-Geisser ε was >0.7, the Huyhn-Feld ε-adjusted F statistic was used to assess significance of the univariate test (F = 6.11, *p* = 0.04, power = 0.86, partial η^2^ = 0.13).

To facilitate the interpretation of findings we re-ran the analyses on untransformed raw latencies and observed virtually the same pattern of results. The time x treatment order interaction was still significant (Huyhn-Feld F = 6.29, *p* = 0.04). This significant time × treatment order interaction was further broken down by simple-effects analyses on 8 × 8 checkerboard latencies within order. A significant main effect of time was observed for the hardware-software training order (*n* = 18, Greenhouse-Geisser F = 4.06, *p* = 0.04). Pairwise comparisons with the Sidak adjustment showed that the P100 response latency at after 5 weeks of training was significantly greater (M = 107.6, SD = 8.94) than the response latency at baseline (M = 104.46, SD = 8.03, *p* = 0.05).

**Figure 4:**
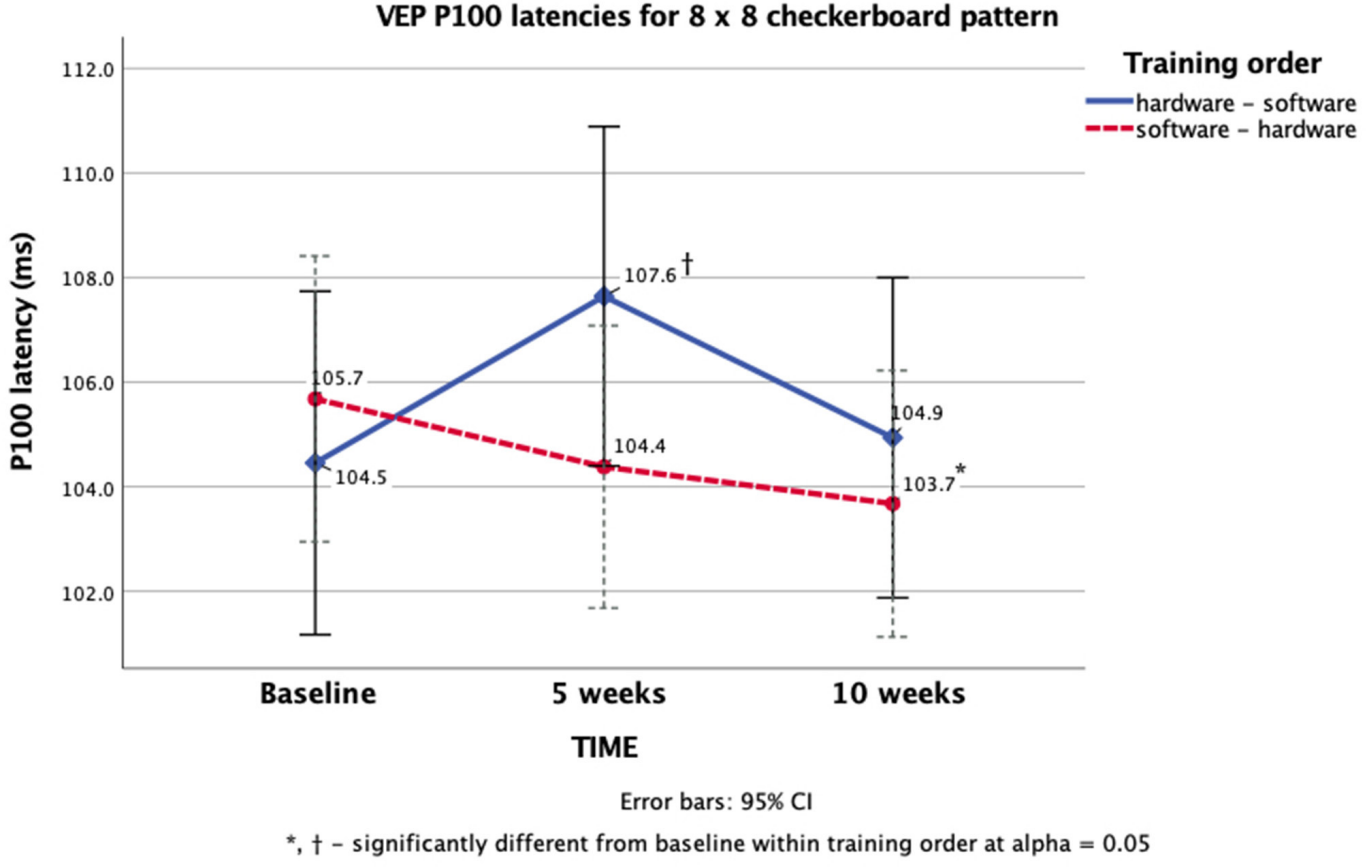
VEP P100 latency differences within training order for the 8 × 8 checkerboard pattern.

A significant main effect of time was also observed for the software-hardware training order (*n* = 26, F = 4.91 [sphericity assumed], *p* = 0.01). Pairwise comparisons with the Sidak adjustment showed that the P100 response latency at after 10 weeks of training was significantly faster (M = 103.67, SD = 5.58) than the response latency baseline (M = 105.68, SD = 6.02, *p* = 0.02). These results are summarized in Figure 4.

#### Amplitudes

There was a significant multivariate effect of time (Wilk's Lambda F = 3.68, *p* < 0.01). This multivariate effect was associated with sufficient statistical power (.96) and a moderate effect size (partial η^2^= 0.46). Neither the effect of order nor the order- by- time interaction were significant.

**Figure 5:**
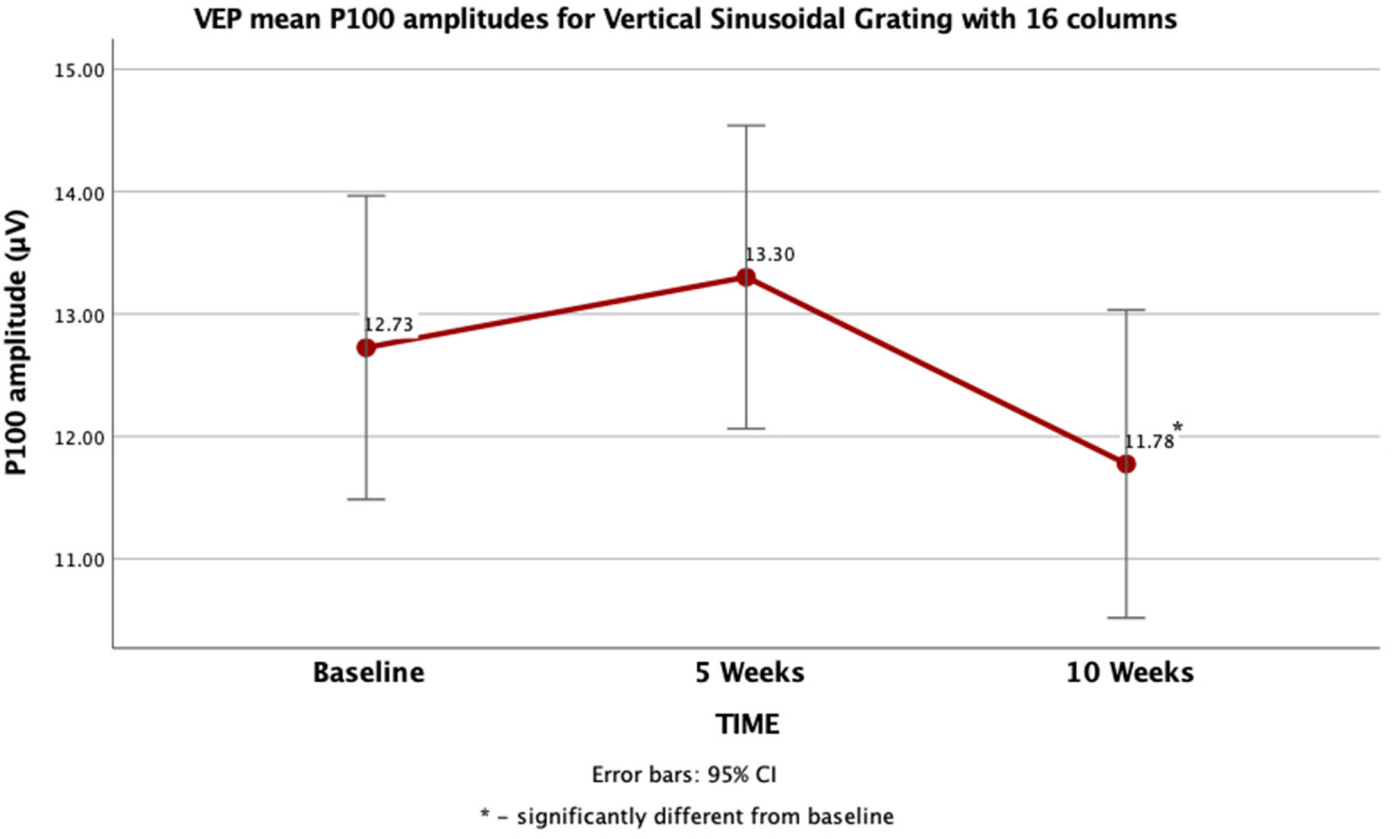
VEP P100 amplitude differences as a function of assessment point in response to a Vertical Sinusoidal Grating with 16 columns.

Univariate analyses revealed that the multivariate significance for the time effect was significant for log10-transformed amplitudes in response to both 16 [*F*_(2, 84)_ = 4.98 *p* < 0.01] and 8-column [*F*_(2, 84)_ = 3.59 *p* = 0.03] vertical sinusoidal gratings but not for checkerboard patterns. Mauchly's test of sphericity for 16 and 8 column vertical sinusoidal gratings was not significant, so sphericity was assumed and no epsilon (ε) adjustment was used. Pairwise comparisons with the Sidak correction showed that for both stimuli sizes log10-transformed amplitudes after 10 weeks of training were significantly lower than after 5 weeks of training at alpha = 0.05. No other comparisons were significant. After re-running the doubly multivariate analysis of variance on raw, untransformed amplitudes, the main effect of time remained for the 16-column vertical sinusoidal grating but not for the 8-column grating. Again, the P100 amplitude at final assessment (M = 11.78, SE = 0.62) was significantly (*p* < 0.01) lower than at the intermediate point (5 weeks: M = 13.30, SE = 0.61). No other pairwise comparisons were significant. These results are summarized in Figure 5.

## Discussion

In the present study we hypothesized that administration of OVT oculomotor training protocols before software training may result in larger performance improvements compared to the reverse order due to the initial strengthening of the visual hardware capable of handling greater demands during training of visuomotor integration and information processing skills (visual software). Within the design of our study that did not include a control group it is difficult to attribute significant post-training improvements observed in both experimental groups on all but one (contrast sensitivity) Nike/Senaptec measures to the actual effects of training. Since some of the software training drills (e.g., dynamic depth perception, eye-hand coordination, decision-making, and split attention) directly mimicked test stimuli, it is of no particular surprise that all athletes improved their performance on the Nike/Senaptec measures by the end of their 10 week visual training. It has also been previously reported that improvements on some of the Nike/Senaptec measures can be explained by practice effects within the test-retest paradigm (Erickson et al., 2011; Liu et al., 2020). We were more interested in a possible treatment order- by- time interaction effect on the Nike/Senaptec measures that could indicate greater utility of a particular vision training approach. While we did not observe this effect on any of the Nike/Senaptec measures, our electroencephalographic findings proved to be more sensitive to the interaction effect and the training effect. This interaction effect was significant for the STMLI measure of accommodative vergence facility (i.e., Near Far Quickness) and for the VEP measure of P100 latency in response to the 8 × 8 checkerboard stimuli with 3.38 cm (2-degree) check size. On these measures significantly lower STMLI ratios and faster P100 latencies were observed after 10 weeks of visual training compared to baseline but only if the athletes started their regimen with 5 weeks of software training first followed by 5 weeks of oculomotor (hardware training). Additionally, both training orders resulted in significant decreases in post-treatment STMLI ratios for perception span, Go/No-Go and Hand Reaction time. Similarly, both treatment orders resulted in significant decreases in P100 amplitude after 10 weeks of training compared to baseline when presented with 0.50 cycle/deg vertical sine waves. Let's discuss these findings and their relevance to performance.

### STMLI Ratios

The interaction effect for the STMLI measures recorded during completion of the Near-Far Quickness test was primarily driven by significant increases in 30–40 Hz Fz gamma power in the software-hardware training group at 5 weeks (M = 2.65, SD = 0.17) and 10 weeks (M = 2.64, SD = 0.17) compared to baseline (M = 2.47, SD = 0.25), and not due to changes in theta power. In the hardware-software training group no significant changes in either theta or gamma band activity was observed compared to baseline. Significant training effects observed in both treatment groups as indexed by lower STMLI ratios post-treatment compared to baseline were also primarily related to increases in the 30–40 Hz Fz gamma power for perception span (M = 2.58, SD = 0.23 vs. M = 2.44, SD = 0.29), Go/No-Go (M = 2.41, SD = 0.26 vs. M = 2.27, SD = 0.22), and hand reaction time (M = 2.35, SD = 0.25 vs. M = 2.26, SD = 0.23). Additionally, on the measure of perception span, lower STMLI ratio was also related to a significantly lower Fz 3–7 Hz theta power after 10 weeks of training (M = 1.19, SD = 0.12) compared to baseline (M = 1.25, SD = 0.15).

In our previous study using STMLI ratios, we showed that this index is a particularly sensitive measure of neuropsychological status (i.e., history of concussion in athletes) especially on tasks of visuomotor control that largely depend on the working memory capacity and temporal processing speeds (Poltavski et al., 2019). Both working memory and timing ability are thought to predict accuracy and quickness of motor responses (Tirre and Raouf, 1998), which are integral components of the Nike/Senaptec tests of near-far quickness, perception span, hand reaction time and Go/No-Go. We previously proposed that the short-term memory load index thus reflects efficiency of the psychomotor ability, i.e., a working memory load under temporal processing constraints on visuo-motor tasks, on which either the presentation of stimulus or the response to a stimulus have a limited time window (Poltavski et al., 2019). In this study changes in STMLI ratios across the above visuo-motor tasks were primarily driven by changes in the gamma band activity.

Gamma oscillations are generated through a feedback mechanism, in which firing of pyramidal cells and the release of glutamate excites a special population of interneurons, which then rapidly inhibit the entire population of pyramidal cells by releasing GABA (Buzsáki, 2001). The source of gamma oscillations has been attributed to the activity these GABAergic interneurons. This mechanism has been later termed “PING,” pyramidal interneuron network gamma (Miller et al., 2018). Since the PING mechanism depends on strong excitation, in the visual cortex increase in oscillatory gamma is directly proportionate to sensory input and stops once the stimulus is terminated (Miller et al., 2018). In the prefrontal cortex, however, external stimulation has less of an impact on overall gamma excitation, which is spikier and more variable and is related to the fact that the PFC integrates inputs from many cortical and subcortical areas (Lundqvist et al., 2016). Miller et al. (2018) proposed a theoretical model, in which encoding and retrieval of information in working memory is associated with bursts of gamma in the prefrontal cortex. Lisman and Jensen (2013) further elaborated that the role of gamma band activity is to define an item in a multi-item message. Specifically, gamma contributions seem to involve facilitation of message encoding by allowing only the most excited cells to fire, synchronization of spikes across cortical regions and parsing of items to prevent errors in decoding the message.

Indeed, increased Fz gamma band activity in the 30–40 Hz range has also been linked to improved performance on short-term and long-term memory tasks and has been suggested to augment feature binding during encoding (Keizer et al., 2010). In the latter study the researchers used a neurofeedback training regimen during which two groups of subjects were encouraged to increase the number of occurrences of an auditory tone that indicated exceeded threshold for the power of 36–40 Hz gamma collected at Oz (GBA+ group) or 12–20 Hz Beta at Oz and Fz (BBA+ group). Following the training the researchers tested whether changes in the target band activity would influence performance on behavioral tasks of short-term and long-term episodic binding. The results showed that across the neurofeedback sessions the GBA+ group was able to significantly increase gamma band activity at the Fz electrode location compared to baseline. On the behavioral level the GBA+ training seemed to have reduced the impact of task-irrelevant feature bindings on performance of both short-term and long-term episodic binding tasks, suggesting enhanced control and management of feature bindings. Additionally, GBA+ training improved accurate recollection of features (i.e., color) of drawings presented during a long-term memory task. The researchers concluded that enhanced gamma band activity at Oz and Fz and increased GBA between frontal and occipital electrode sites allows for a greater flexibility in handling integrated information in both short-term and long-term memory.

Significant reduction in the frontal theta band activity (TBA) in our study during the completion of the perception span task is also consistent with proposed theoretical models for the role of theta as well as with empirical evidence. Besides its organizing role in working memory formation as a phase reference for multi-item messages (Lisman and Jensen, 2013), frontal (Fz) theta band activity has been implicated in cognitive workload, recruitment of cortical resources and performance of complex cognitive tasks (Doppelmayr et al., 2008; Borghini et al., 2014). Borghini et al. (2013) tested changes in performance of novice pilots on a flight simulation task and a Multi Attribute Task Battery test (MATB) after 5 sessions of daily practice. Significantly improved performance on both tasks in the end of the training protocol compared to baseline was also accompanied by significant reduction of theta PSD over the frontal midline channel Fz. This reduction correlated with similar reductions in heart rate and eye-blink-responses as well as perceived workload. The researchers concluded that frontal theta may be useful in monitoring training progress as an index of cognitive resource recruitment and information processing. The utilization of a measure of both band activities in the form of the frontal theta-to-gamma amplitude ratio thus seems to offer a sensitive index of a learning process. Reductions in STMLI ratios appear to suggest greater efficiency in feature encoding and retrieval, suppression of irrelevant information and cognitive resource allocation. This EEG profile is further associated with improved performance on visuo-motor tasks of the Nike/Senaptec Sensory stations.

### VEP P100

We observed a significant post-training reduction of the P100 latency in the software-hardware training group in response to checkerboard stimuli of 2° checks presented with 85% contrast and the temporal frequency of 1 Hz (2 reversals per second). The finding of reduced P100 latencies to this type of stimulus following visual training of athletes in itself is not novel and is consistent with previous studies. First, several research groups previously showed that athletes participating in dynamic, fast-paced sports (e.g., volleyball, tennis, squash, fencing and karate) show faster P100 latencies compared to non-athletes in response to similar checkerboard stimuli (Taddei et al., 1991; Del Percio et al., 2007; Delpont et al., 1991; Zwierko et al., 2010), which is thought to be directly dependent on the visual processing requirements of their sport and explain their faster reaction times on reaction time tests compared to controls (e.g., Zwierko et al., 2010; Hülsdünker et al., 2018). Second, sports-specific training has also been showed to further reduce P100 latencies in these athletes possibly improving their early sensory processing and optimizing visual attention the relevant stimuli (Zwierko et al., 2014). The novelty of our findings is related to the observation of this latency reduction only in the software-hardware training group, although both groups received identical visual training procedures just in different temporal order. The implications of this observation will be discussed below in the section on training order.

On the other hand, the hardware-software training group showed a transient significant increase in the P100 latency after 5 weeks of oculomotor training before going back to normal at the end of the 10 weeks. We did not find any studies using pattern VEP stimuli with normal subjects undergoing oculomotor training. Furthermore, when Yadav et al. (2014) administered 9 h of oculomotor rehabilitation protocols to adult individuals with a history of mTBI, they only reported increases in their P100 amplitudes but no changes in their P100 latencies. The observed results in the present study may indicate an unexpected interaction of the type of treatment with developmental factors and gender. In a study of 406 normal subjects, 6–80 years of age, Emmerson-Hanover et al. (1994) reported that in general pattern-reversal VEP latencies were found to decrease during maturation, stabilize across early adulthood, then begin to increase sometime after the late 20s. Although there were minimal gender differences in latencies during development, males in that study tended to have longer latencies than females during adulthood. The effects of optometric vision training protocols in normal populations including athletes should be studied further across different age groups to understand the implication of this finding further.

Another original finding in the present study was an observed reduction of the P100 amplitude after 10 weeks of training compared to baseline in response to 0.50 cycle/deg vertical sine wave gratings pattern presented with 10% contrast and reversed at a rate of 4 rev/s (2 Hz). This decrease did not depend on the training order. On the surface these results may seem counterintuitive. For example, in clinical populations (e.g., mTBI patients, amblyopia) administration of vision therapy resulted in increased P100 amplitude suggesting greater activation of the visual cortex that resulted in improved performance on tests of visual attention and visual acuity, respectively (Oner et al., 2004; Yadav et al., 2014). Similarly, Bao et al. (2010) reported increased VEP amplitudes in normal subjects following several weeks of perceptual training. There are at least 2 issues that should be considered here. First, is normalization of cortical function in clinical populations equivalent to optimization of cortical function in visually trained athletes? The second issue has to do with the component of a sinusoidal VEP waveform that is the deflection of interest in a specific perceptual learning study. The importance of the latter question is related to differences in stages in visual processing and involvement of different neuronal populations. For example, in the study by Bao et al. (2010) the researchers were focusing on the changes in the amplitude of the C1 VEP components in normal subjects following several weeks of training to detect a diagonal grating pattern in one of the 4 quadrants of the visual field. C1 is considered the earliest VEP component that peaks around 50–70 ms after stimulus presentation and reverses polarity depending whether the upper or the lower visual field is stimulated. This is taken as evidence that it is primarily generated in V1 (Bao et al., 2010). The researchers attributed the increase in their C1 amplitude following perceptual training to induced plasticity in early visual cortex through local receptive field changes that facilitated signal boosting.

At the same time Casco et al. (2004) reported P1 (same as P100) amplitude decrease and N1 (same as N75) amplitude increase in a learning paradigm where normal subjects had to judge the orientation of texture bar appearing in different configurations against a uniform texture background. This learning related change in VEP component amplitudes was associated with significantly increased performance accuracy. The researchers suggested that the effect of P1 is likely related to response preparation, whereas N1 likely represents global texture segmentation. Specifically, Casco et al. (2004) argued the effect of an increased performance accuracy associated with P100 amplitude attenuation suggests inhibition of the texture orientation conflicting with the orientation against which the orientation of the bar is judged. The P1 (or P100) amplitude decrease is thus linked to inhibition of an irrelevant orientation configuration. Similarly, in the present study the attenuation of the P100 amplitude component after 10 weeks of visual training compared to baseline may be related to more efficient inhibition of irrelevant visual information. During our VEP recording subjects are instructed to focus on a small red rotating annular fixation cross in the center of the screen during presentation of reversing checkerboard or vertical sinusoidal grating stimuli. These instructions are thought to help to control accuracy of fixation and accommodation as well as to maintain visual attention. Additionally, Shete et al. (2019) recently reported reduced P100 amplitudes in male volleyball players compared to age-matched controlled. The researchers similarly suggested improved efficiency of early visual processing in the athletic group due to rapid visual processing demands of their sport.

Nonetheless, the P100 amplitude reduction in our study was observed in response to a transient (magnocellular) stimulus and not in response to sustained parvocellular stimuli (checkerboard patterns). The magnocellular system may, according to Laycock et al. (2008) may be involved in coordination of attentional processing and attentional guidance. In studies with healthy human participants the latency difference in P100 response to magnocellular stimuli is 20–30 ms faster than to parvocellular stimuli, a finding known as “magnocellular advantage” (Laycock et al., 2008). The activation of the magnocellular pathway before the parvocellular pathway has been suggested to involve the dorsal stream and area MT/V5 in a feedforward mechanism that is thought to help shift and guide attentional focus maintained by the parvocellular system and the ventral stream (Laycock et al., 2007, 2008).

This elegant narrative, however, is somewhat muddled by the absence of the magnocellular advantage in the present study. In fact, on average, participants in both groups were about 13 ms slower in their response to magnocellular (VSG) stimuli than to parvocellular (checkerboard stimuli). Part of the reason may be related to undiagnosed/unreported history of concussion in this group of young ice hockey players. Although we did remove from the analyses the data from the players who had reported a history of concussion, it is very possible that within such a traumatic sport as ice hockey many of the concussions remain undiagnosed and underreported. This would certainly fit with the VEP profile of the athletes in our study, as we previously showed that individuals with a history of concussion had significantly slower response latencies to magnocellular stimuli than those without such history (Poltavski et al., 2017). Nonetheless, the results of the present study suggest changes in the engagement of the magnocellular system at the end of the training in response to early visual processing stimuli, which would be consistent with Laycock et al. (2007, 2008) model of the parvocellular-ventral attentional system guidance by the magnocellular—dorsal pathway.

### Training Order

Contrary to our original hypothesis, software training before oculomotor training showed more favorable electrophysiological profiles consistent with more efficient visual processing. Our hypothesis was largely based on the results of a series of studies by the Ciuffreda group that showed that administration of oculomotor vision training emphasizing vergence, accommodation and version to individuals with a history of mTBI resulted not only in significant clinical improvements of their oculomotor deficits (Thiagarajan and Ciuffreda, 2013, 2014, 2015; Thiagarajan et al., 2014) but also in normalization of their VEP profiles and improved scores on the visual search and attention Tests (Yadav et al., 2014). These authors attributed observed improvements to stabilization of vergence and accommodation. Nonetheless, in a recent review Barton and Ranalli (2020) referenced a Cochrane review to suggest that the evidence pertaining to the efficacy of oculomotor training in the above studies had a “very low certainty.” Some of the specific points of criticism included very small sample sizes, fairly high drop-out rates (between 25 and 45%) as well as the use of a rapid serial visual presentation task during training sessions, a technique that, according to the authors, clearly requires sustained attention, concentration and working memory. Barton and Ranalli (2020) thus contended that observed functional improvements in the mTBI group on the clinical Visual Search and Attention Test (VSAT) in the Yadav et al. (2014) study could be attributed to inadvertent training of attention rather than eye movements. The authors further noted that one of the most common oculomotor deficits in mTBI related to version, such as visually guided saccades, typically do not appear anomalous until the patient is placed under conditions of increased cognitive workload with working memory and attentional demands (i.e., antisaccades and memory guided saccades). They cited the study by Heitger et al. (2009) to conclude that oculomotor problems in mTBI may be secondary to observed cognitive issues with decision making, attention, sequence programming and response inhibition. It is thus possible that training of information processing skills first is also likely to affect some oculomotor skills but do so in a less perceptually stressful and more functionally compound context as opposed to isolated and targeted oculomotor training often seen in OVT.

Indeed, Caldani et al. (2020) recently reported that children with ADHD and poor pursuit eye movements were able to significantly decrease the number of catch-up saccades following visual-attentional training with serial search tasks. In the Sports Vision Training realm Appelbaum et al. (2016) showed that application of traditional SVT drills to collegiate athletes resulted in significant improvements in at least two sensorimotor skills on the Sensory Station: accommodative vergence facility (i.e., Near Far Quickness) and dynamic visual acuity (i.e., Target Capture). We thus propose that in the present study our visual software training procedures also indirectly trained the oculomotor system. This effect may have better prepared our athletes for more targeted, isolated and fairly intense exercises of the orthoptics arm of our visual training regimen.

### Conclusions, Limitations, and Future Research Implications

Overall, the results of the present study suggest that our comprehensive training of the oculomotor system in combination with training of the visual information processing skills resulted in significant improvements on most measures of the Sensory Station. More importantly we were able to show the utility of the EEG-derived STMLI ratios to monitor training progress. This index suggested greater efficiency in visual information processing and cognitive resource allocation following 10 weeks of visual training. Similarly, our VEP components of P100 latency and amplitude also suggested improved speeds of visual signal processing at the end of study in combination with more effective and parsimonious cognitive resource allocation and attentional engagement. These electrophysiological indexes in some cases favored the software-hardware training order of procedural administration. This may suggest improved preparedness of the oculomotor system in our youth athletes for administration of targeted protocols of the Optometric Vision Therapy. Future studies with athletes and comprehensive SVT protocols should also include a placebo control group, and should also compare the effectiveness of such training in athletes with a history of concussion. Convenience sampling, age variability, developmental factors and correlational nature of the findings observed in the present study may further limit their generalizability onto athletic populations and, therefore, require further inquiry. Pre- and post-training measures should also include direct measures of athletic performance to evaluate the degree of transfer on the actual performance in the sport of interest. Both EEG and VEP measures used in the present study have considerable potential for future utilization in SVT research and clinical populations.

## Data Availability Statement

The raw data supporting the conclusions of this article will be made available by the authors, without undue reservation.

## Ethics Statement

The studies involving human participants were reviewed and approved by the Institutional Review Board of the University of North Dakota. Written informed consent to participate in this study was provided by the participants' legal guardian/next of kin.

## Author Contributions

DP oversaw all aspects of the study including subject recruitment, testing, training, and data collection. He conducted all statistical analyses and wrote up the bulk of the manuscript sections. DB significantly contributed to subject recruitment, testing, data collection, analysis interpretation, and literature review. CP significantly contributed to subject testing and training as well as to the preparation of the materials and procedures sections of the manuscript. All authors contributed to the article and approved the submitted version.

## Funding

This study was funded by the North Dakota Department of Commerce: Research ND Program. Project title: Evaluation of the Effectiveness of Sports Vision Programs in Improving Performance and Health in ND Youth Athletes. Award Dates: 2015–2017. Award # 15-02-G-88.

## Conflict of Interest

DB was employed by the company Valley Vision Clinic. The remaining authors declare that the research was conducted in the absence of any commercial or financial relationships that could be construed as a potential conflict of interest.

## Publisher's Note

All claims expressed in this article are solely those of the authors and do not necessarily represent those of their affiliated organizations, or those of the publisher, the editors and the reviewers. Any product that may be evaluated in this article, or claim that may be made by its manufacturer, is not guaranteed or endorsed by the publisher.
